# RELATIONSHIP BETWEEN TIMING OF SURGERY AFTER PREOPERATIVE BILIARY DRAINAGE AND OUTCOMES FOLLOWING PANCREATODUODENECTOMY

**DOI:** 10.1590/S0004-2803.24612026-050

**Published:** 2026-10-30

**Authors:** Philipe Franco do Amaral TAFNER, Bruna Mattos Vanetti de ALBUQUERQUE, Fernando Girardi da SILVA, Martinho Antonio GESTIC, Murillo Pimentel UTRINI, Francisco CALLEJAS-NETO, Everton CAZZO

**Affiliations:** * Universidade Estadual de Campinas (UNICAMP), Faculdade de Ciências Médicas, Departamento de Cirurgia, Campinas, SP, Brasil.

**Keywords:** Pancreaticoduodenectomy, biliary tract surgical procedures, cholestasis, extrahepatic, drainage, postoperative complications, Pancreatoduodenectomia, procedimentos cirúrgicos do sistema biliar, colestase extra-hepática, drenagem, complicações pós-operatórias

## Abstract

**Background:**

The role and optimal timing of surgery following preoperative biliary drainage (PBD) in patients undergoing pancreatoduodenectomy remain controversial. Early surgery may be influenced by procedure-related inflammation, whereas delayed intervention may increase infectious complications due to biliary colonization.

**Objective:**

To evaluate the impact of the interval between biliary drainage and pancreatoduodenectomy on postoperative outcomes and its association with biliary contamination.

**Methods:**

This retrospective cohort study included 239 patients undergoing pancreatoduodenectomy between 2013 and 2025. Of these, 174 (73%) underwent PBD via endoscopic retrograde cholangiopancreatography. Patients were stratified by interval between drainage and surgery: ≤2 weeks, 3-6 weeks, and >6 weeks. The primary outcome was 90-day mortality; secondary outcomes included major complications (Clavien-Dindo ≥III). Multivariable logistic regression adjusted for confounders.

**Results:**

Surgery at 3-6 weeks was associated with the lowest rates of major complications (31.7%) and 90-day mortality (9.7%), compared with ≤2 weeks (57.1%; 28.6%) and >6 weeks (40.6%; 23.2%). Surgery outside this interval increased 90-day mortality risk (OR 2.9, 95%CI 1.07-7.83). Biliary colonization rose with longer intervals, exceeding 90% after three weeks.

**Conclusion:**

A 3-6 week interval between biliary drainage and pancreatoduodenectomy is associated with improved outcomes, suggesting an optimal window balancing inflammation and bacterial colonization.

## HIGHLIGHTS

• Pancreatoduodenectomy timing after biliary drainage influences postoperative outcomes.

• Surgery at 3-6 weeks was associated with the lowest major complication rate.

• Ninety-day mortality was lowest when surgery was performed 3-6 weeks after drainage.

• Surgery outside the 3-6-week window was associated with increased 90-day mortality.

• Biliary colonization increased markedly with longer intervals between drainage and surgery.

## RESUMO

**Contexto:**

O papel e o momento ideal para a realização da cirurgia após a drenagem biliar pré-operatória (DBP) em pacientes submetidos à pancreatoduodenectomia permanecem controversos. A cirurgia precoce pode ser influenciada pelo processo inflamatório relacionado ao procedimento, enquanto o adiamento da intervenção pode aumentar as complicações infecciosas em decorrência da colonização biliar.

**Objetivo:**

Avaliar o impacto do intervalo entre a drenagem biliar e a pancreatoduodenectomia nos desfechos pós-operatórios e sua associação com a contaminação biliar.

**Métodos:**

Este estudo de coorte retrospectivo incluiu 239 pacientes submetidos à pancreatoduodenectomia. Destes, 174 (73%) foram submetidos à DBP por colangiopancreatografia retrógrada endoscópica. Os pacientes foram estratificados de acordo com o intervalo entre a drenagem e a cirurgia: ≤2 semanas, 3-6 semanas e >6 semanas. O desfecho primário foi a mortalidade em 90 dias, e os desfechos secundários incluíram complicações maiores (Clavien-Dindo ≥III). Foi realizada regressão logística multivariada com ajuste para potenciais fatores de confusão.

**Resultados:**

A realização da cirurgia entre 3 e 6 semanas após a drenagem esteve associada às menores taxas de complicações maiores (31,7%) e de mortalidade em 90 dias (9,7%), em comparação com a cirurgia realizada em ≤2 semanas (57,1% e 28,6%, respectivamente) ou após >6 semanas (40,6% e 23,2%, respectivamente). A realização da cirurgia fora do intervalo de 3-6 semanas esteve associada a um risco 2,9 vezes maior de mortalidade em 90 dias (OR 2,9; IC95% 1,07-7,83). A colonização biliar aumentou com o prolongamento do intervalo até a cirurgia, ultrapassando 90% após três semanas.

**Conclusão:**

O intervalo de 3-6 semanas entre a drenagem biliar e a pancreatoduodenectomia esteve associado a melhores desfechos pós-operatórios, sugerindo uma possível janela ideal que equilibre a resolução do processo inflamatório relacionado ao procedimento e a progressão da colonização bacteriana.

## INTRODUCTION

Pancreatoduodenectomy remains the only potentially curative treatment for malignant tumors arising from the pancreatic head and periampullary region. However, these tumors frequently present with obstructive jaundice, which may result in impaired hepatic function, coagulation abnormalities, renal dysfunction, and increased susceptibility to infection. For these reasons, preoperative biliary drainage (PBD) is commonly performed in patients with significant biliary obstruction prior to definitive surgical treatment^1^ ^,^ ^2^.

Endoscopic retrograde cholangiopancreatography (ERCP) with stent placement is currently the most widely used technique for biliary decompression. Nevertheless, the role of preoperative biliary drainage remains controversial^3^ ^-^ ^5^. While biliary decompression may improve physiological status in patients with severe jaundice, several studies have demonstrated that PBD can also increase postoperative infectious complications. This effect has been attributed primarily to bacterial colonization of the biliary tree and biofilm formation on biliary stents, which may lead to polymicrobial contamination of bile and intraoperative bile spillage^3^ ^-^ ^5^.

In addition to the question of whether drainage should be performed, an important but less clearly defined issue concerns the optimal timing of surgery following biliary decompression. Early surgery after ERCP may be associated with inflammatory changes caused by endoscopic manipulation, potentially increasing operative difficulty and postoperative morbidity^6^. Conversely, delaying surgery may allow progressive colonization of the biliary tract, increasing the risk of infectious complications due to bacterial biofilm development on indwelling stents^7^.

These opposing mechanisms suggest that the interval between biliary drainage and surgery may have a meaningful impact on postoperative outcomes. However, existing studies addressing this issue have produced inconsistent results, and there is currently no consensus regarding the optimal interval between biliary drainage and pancreatoduodenectomy^7^ ^,^ ^8^. Moreover, the relationship between surgical timing, biliary colonization, and postoperative complications remains incompletely understood^9^.

Therefore, the aim of the present study was to evaluate the impact of the interval between biliary drainage and pancreatoduodenectomy on postoperative outcomes in a large cohort of patients undergoing pancreatic surgery. In addition, we sought to investigate the relationship between surgical timing, biliary contamination, and postoperative morbidity, with the goal of identifying a potentially optimal interval between biliary drainage and surgery. We hypothesized that an intermediate interval between biliary drainage and surgery would be associated with lower postoperative morbidity and mortality compared with very early or delayed surgical intervention.

## METHODS

### Study design and population

This retrospective cohort study included consecutive patients who underwent pancreatoduodenectomy at a tertiary referral center between January 2013 and October 2025. Patients undergoing resection for malignant or premalignant periampullary tumors were eligible for inclusion. Patients were identified from a prospectively maintained institutional database, and clinical, perioperative, pathological, and follow-up data were systematically reviewed. Patients without available information regarding preoperative biliary drainage or the interval between drainage and surgery were excluded. This study was approved by the institutional review board (IRB approval no. 8.318.753; CAAE 73914923.7.0000.5404) and conducted in accordance with the Declaration of Helsinki.

### Preoperative biliary drainage and clinical decision-making

Preoperative biliary drainage was performed using endoscopic retrograde cholangiopancreatography with biliary stent placement. The indication for drainage was determined by a multidisciplinary team based on clinical presentation, including the severity of jaundice, presence of cholangitis, impaired hepatic function, and the anticipated need to delay surgery. The timing of surgery following biliary drainage was not standardized and reflected real-world clinical practice, being influenced by a combination of patient-related factors such as clinical condition, nutritional status, and performance status; disease-related factors including tumor stage and severity of biliary obstruction; treatment-related considerations such as the use of neoadjuvant therapy and response to biliary decompression; as well as institutional and logistical factors including referral patterns and operating room availability. As a result, patients undergoing early or delayed surgery may have differed systematically in baseline characteristics, introducing the potential for confounding by indication.

### Data collection

Detailed clinical variables were collected to characterize patient status at the time of surgical decision-making and to address potential confounding. These included demographic characteristics such as age, sex, and body mass index, as well as comorbidities, ASA classification, and ECOG performance status. Laboratory parameters were recorded, including preoperative bilirubin levels, and the presence of cholangitis at presentation was documented. Tumor-related variables included histological diagnosis and stage, while treatment-related factors included the use of neoadjuvant therapy, the number of ERCP procedures performed, and the type of biliary stent used. Operative variables such as operative time, estimated blood loss, vascular resection, and transfusion requirements were also recorded.

### Timing of surgery after biliary drainage

Patients undergoing preoperative biliary drainage were stratified according to the interval between the last ERCP and surgery into three predefined groups: those undergoing surgery within two weeks, those operated between three and six weeks, and those undergoing surgery after more than six weeks. These intervals were selected a priori based on biological and clinical considerations, with the early interval intended to capture the immediate post-ERCP inflammatory phase, the delayed interval reflecting prolonged stent dwell time and increased likelihood of biliary colonization, and the intermediate interval representing a potential balance between these competing processes. Given the anticipated imbalance in group sizes, particularly a smaller number of patients in the early interval group, a secondary analysis was performed comparing patients undergoing surgery within the three- to six-week interval with all other patients combined, in order to improve statistical robustness and to further explore the clinical relevance of this intermediate time window. Group sizes for each interval were explicitly reported to allow assessment of distribution and potential imbalance.

### Postoperative outcomes

The primary outcome of the study was 90-day mortality. Secondary outcomes included major postoperative complications, defined as Clavien-Dindo grade III or higher, as well as specific complications such as postoperative pancreatic fistula, delayed gastric emptying, surgical site infection, postoperative bleeding, and the need for reoperation. Postoperative pancreatic fistula and delayed gastric emptying were defined according to established international criteria, and mortality was assessed at 30, 60, and 90 days following surgery.

### Biliary microbiology

In patients undergoing preoperative biliary drainage, intraoperative bile samples were obtained for microbiological culture when available. The presence of bacteriobilia and polymicrobial flora was recorded, and the proportion of patients with available bile cultures in each timing group was documented to account for potential sampling bias.

### Statistical analysis

Continuous variables were expressed as mean with standard deviation (SD) or as median with interquartile range (IQR), depending on data distribution, while categorical variables were presented as counts and percentages. Comparisons between groups were performed using the chi-square test or Fisher’s exact test for categorical variables and appropriate parametric or non-parametric tests for continuous variables.

To address potential confounding by indication, a multivariable logistic regression model was constructed to identify independent predictors of 90-day mortality. Variables considered clinically relevant and those with a *p-*value <0.25 in univariate analysis were included in the multivariable model. These variables comprised demographic characteristics, baseline performance status, indicators of disease severity, oncological features, treatment-related factors, and the interval between biliary drainage and surgery. Odds ratios with 95% confidence intervals were calculated, and a two-sided *p*-value <0.05 was considered statistically significant. Statistical analyses were performed using SAS System for Windows version 9.4 (SAS Institute Inc., Cary, NC, USA) and R version 4.2.2 (The R Foundation for Statistical Computing).

## RESULTS

### Patient characteristics

A total of 174 patients undergoing pancreatoduodenectomy with prior ERCP were included in the study. The mean age was 60.8 ± 10.9 years, and 54% were female. The mean body mass index was 24.4 ± 4.2 kg/m². Most patients presented with relevant comorbidity, with 50.6% classified as ASA III. Regarding performance status, 65% of patients had ECOG 0-1.

All individuals underwent biliary drainage through endoscopic retrograde cholangiopancreatography. The mean number of ERCP procedures was 1.19 ± 0.51. Plastic stents were the most commonly used devices (65%), whereas 10% of the patients received metallic stents. In the remaining patients, the type of stent could not be identified from the medical records. Among all included individuals, 139 had the interval between ERCP and surgery available in the medical records. The median interval between biliary drainage and surgery was 42 days (IQR 30 - 64). Intraoperative bile cultures were available in 146 of 174 patients (83.9%) undergoing preoperative biliary drainage, corresponding to 61.1% of the overall cohort.

The most frequent histological diagnosis was pancreatic ductal adenocarcinoma (39%), followed by ampullary carcinoma (28%) and distal bile duct carcinoma (10%). Tumor stage distribution included 31% stage IIB and 20% stage III disease.

Table 1 summarizes the main characteristics of the study population.

**TABLE 1:** Baseline characteristics of individuals undergoing pancreatoduodenectomy following preoperative biliary drainage.

Variable	Value
**N**	**239**
Age (years)	60.8 ± 10.9
BMI (kg/m²)	24.4 ± 4.2
Female sex	129 (54%)
Male sex	110 (46%)
ASA III	121 (50.6%)
ECOG 0-1	154 (65%)
Preoperative biliary drainage	174 (73%)
**ERCP characteristics**	
**N**	**174**
Number of ERCP procedures	1.19 ± 0.51
Plastic stent	113 (65%)
Metal stent	18 (10%)
Interval ERCP → surgery (days)	68.6 ± 122.8

N: number of individuals. BMI: body mass index. ASA: American Society of Anesthesiologists physical status classification. ECOG: eastern cooperative oncology group performance status. ERCP: endoscopic retrograde cholangiopancreatography.

### Perioperative outcomes

The mean operative time was 315.2 ± 57.6 minutes, with a mean estimated blood loss of 1025.2 ± 747 ml. The mean intensive care unit stay was 7.7 ± 11.8 days, and the mean total hospital stay was 18.9 ± 18.3 days.

Postoperative pancreatic fistula occurred in 45 patients (18.8%), delayed gastric emptying in 41 patients (17.2%), and surgical site infection in 29 patients (12.1%). Reoperation was required in 39 patients (16.3%), and postoperative bleeding occurred in 18 patients (7.5%). Major postoperative complications (Clavien-Dindo ≥III) occurred in 89 patients (37.2%). In-hospital mortality rate was 14.6% (Table 2).

**TABLE 2:** Perioperative outcomes of individuals undergoing pancreatoduodenectomy following preoperative biliary drainage.

Outcome	Value
Operative time (min)	315.2 ± 57.6
Blood loss (ml)	1025.2 ± 747
ICU stay (days)	7.7 ± 11.8
Hospital stay (days)	18.9 ± 18.3
**Postoperative complications**	
**Complication**	**N (%)**
Pancreatic fistula	45 (18.8%)
Delayed gastric emptying	41 (17.2%)
Surgical site infection	29 (12.1%)
Postoperative bleeding	18 (7.5%)
Reoperation	39 (16.3%)
**Outcome**	**N (%)**
Major complications (Clavien ≥III)	89 (37.2%)
30-day mortality	35 (14.6%)
60-day mortality	44 (18.4%)
90-day mortality	46 (19.2%)

N: number of patients. min: minutes. ml: milliliters. ICU: intensive care unit. Clavien ≥III: complications grade III or higher according to the Clavien-Dindo classification.

### Timing of surgery after biliary drainage

The groups were generally comparable regarding age, comorbidities, oncological features, and laboratory parameters. Female sex was more frequent in the ≤2-week group (*p*=0.024), while positive bile cultures were significantly more common among patients undergoing surgery after more than 2 weeks from biliary drainage (p<0.001). Among the immediate preoperative laboratory parameters, only INR differed significantly between groups (p=0.015). Baseline demographic, clinical, pathological, and laboratory characteristics according to the interval between biliary drainage and surgery are summarized in Table 3.

**TABLE 3:** Comparison of characteristics of individuals undergoing pancreatoduodenectomy following biliary drainage according to the time elapsed between drainage and surgery.

Variable	≤2 weeks (n=7)	3-6 weeks (n=63)	>6 weeks (n=69)	***P*-value**
Age, mean ± SD (years)	63.4 ± 11.6	61.3 ± 10.5	61.0 ± 10.6	0.664
Female sex, n (%)	6 (85.7)	25 (39.7)	39 (56.5)	0.024
BMI, mean ± SD (kg/m²)	24.1 ± 2.9	25.3 ± 4.8	23.5 ± 4.2	0.193
ASA ≥3, n (%)	2 (28.6)	29 (46.0)	42 (60.9)	0.x100
ECOG ≥2, n (%)	3 (42.9)	17 (27.4)	26 (37.7)	0.397
Stage III/IV disease, n (%)	2 (33.3)	23 (41.1)	16 (26.7)	0.260
Positive bile culture, n (%)	3 (42.9)	59 (100.0)	65 (95.6)	<0.001
Polymicrobial bile culture, n (%)	1 (33.3)	42 (73.7)	51 (79.7)	0.164
**Pathological diagnosis**				
Pancreatic ductal adenocarcinoma, n (%)	4 (57.1)	33 (53.2)	28 (41.8)	0.38
Ampullary adenocarcinoma, n (%)	2 (28.6)	20 (32.3)	19 (28.4)	
Distal cholangiocarcinoma, n (%)	0 (0)	5 (8.1)	10 (14.9)	
Other histologies, n (%)	1 (14.3)	4 (6.5)	10 (14.9)	
**Admission laboratory values**				
Hemoglobin (g/dL)	12.4 [10.8-14.1]	12.2 [10.6-13.8]	12.5 [10.6-13.5]	0.963
Platelets (×10^6^/L)	262 [173.5-446]	286 [219-387]	290.5 [233-375.5]	0.226
Creatinine (mg/dL)	0.8 [0.7-1.1]	0.8 [0.6-0.9]	0.8 [0.6-0.9]	0.140
INR	1.0 [0.9-1.1]	1.1 [1.0-1.3]	1.1 [1.0-1.3]	0.597
Albumin (g/dL)	4.1 [3.8-4.4]	3.7 [3.2-4.2]	3.6 [3.2-4.2]	0.922
AST (U/L)	128 [89-288]	96 [49-181]	89 [45-162]	0.384
ALT (U/L)	254 [197-291]	138 [77-254]	126 [61-214]	0.407
ALP (U/L)	230 [199-1783]	489 [244-1013]	450 [223-881]	0.495
GGT (U/L)	736 [724-1309]	634 [266-1085]	578 [250-1041]	0.888
Total bilirubin (mg/dL)	3.1 [2.4-12.2]	5.8 [2.6-13.0]	5.5 [2.5-10.8]	0.836
Direct bilirubin (mg/dL)	2.5 [2.1-10.0]	4.8 [2.1-10.2]	4.3 [1.9-8.7]	0.917
Glucose (mg/dL)	189.5 [138-291]	104 [92-132]	104 [92-126]	0.058
CA 19-9 (U/mL)	281 [92-414]	171 [38-530]	175 [41-668]	0.865
**Immediate preoperative laboratory values**				
Hemoglobin (g/dL)	10.9 [10.0-12.9]	12.0 [10.7-13.4]	12.8 [10.8-13.9]	0.073
Platelets (×10^6^/L)	320 [233-396]	265 [205-350]	256 [206.8-322.8]	0.085
Creatinine (mg/dL)	0.7 [0.6-0.8]	0.7 [0.6-0.9]	0.7 [0.6-0.9]	0.180
INR	1.0 [1.0-1.0]	1.0 [1.0-1.1]	1.0 [1.0-1.1]	0.015
Albumin (g/dL)	4.0 [3.4-4.1]	3.9 [3.4-4.3]	3.9 [3.5-4.2]	0.135
AST (U/L)	21 [15-73]	24 [17-43]	22 [15-35]	0.755
ALT (U/L)	23 [20-23]	28 [18-57]	28 [16-45]	0.863
ALP (U/L)	599 [257-1706]	284 [161-537]	239 [150-445]	0.061
GGT (U/L)	431 [340-1270]	323 [128-655]	241 [103-511]	0.145
Total bilirubin (mg/dL)	1.1 [0.3-4.7]	1.0 [0.5-2.0]	0.7 [0.4-1.5]	0.266
Direct bilirubin (mg/dL)	1.8 [1.8-3.9]	0.7 [0.3-1.3]	0.4 [0.2-0.9]	0.091
Glucose (mg/dL)	99 [98-106]	98 [89-121]	102 [92-120]	0.794
CA 19-9 (U/mL)	266 [123-409]	126 [33-387]	113 [31-334]	0.756

Values are presented as mean ± standard deviation, median [interquartile range], or number (%), as appropriate. Immediate preoperative laboratory values refer to tests collected on the day prior to surgery. BMI: body mass index. ASA: American Society of Anesthesiologists physical status classification. ECOG: Eastern Cooperative Oncology Group performance status. INR: international normalized ratio. AST: aspartate aminotransferase. ALT: alanine aminotransferase. ALP: alkaline phosphatase. GGT: gamma-glutamyl transferase. CA 19-9: carbohydrate antigen 19-9. Other histologies included rare pancreatic and periampullary neoplasms. Statistically significant *P*-values are shown in bold.

The lowest rate of major complications was observed in patients undergoing surgery 3-6 weeks after drainage (31.7%), compared with 57.1% in patients operated within 2 weeks and 40.6% in those operated after more than 6 weeks. Similarly, 90-day mortality was lowest in the 3-6 week group (9.7%), compared with 28.6% in the ≤2 week group and 23.2% in the >6 week group (Table 4).

**TABLE 4:** Outcomes according to interval between biliary drainage and surgery in individuals undergoing pancreatoduodenectomy following preoperative biliary drainage.

Interval	N	Major complications	90-day mortality
≤2 weeks	7	4 (57.1%)	2 (28.6%)
3-6 weeks	63	20 (31.7%)	6 (9.7%)
>6 weeks	69	28 (40.6%)	16 (23.2%)

N: number of patients.

### Biliary colonization according to surgical timing

The prevalence of positive bile cultures increased with longer intervals between drainage and surgery. Biliary colonization was present in 42.9% of patients operated within 2 weeks, compared with 93.7% in those operated between 3-6 weeks and 94.2% in patients operated after more than 6 weeks. Similarly, polymicrobial flora was observed in 14.3%, 66.7%, and 73.9% of patients in the ≤2 week, 3-6 week, and >6 week groups, respectively (Figures 1, 2 and 3).

**FIGURE 1:**
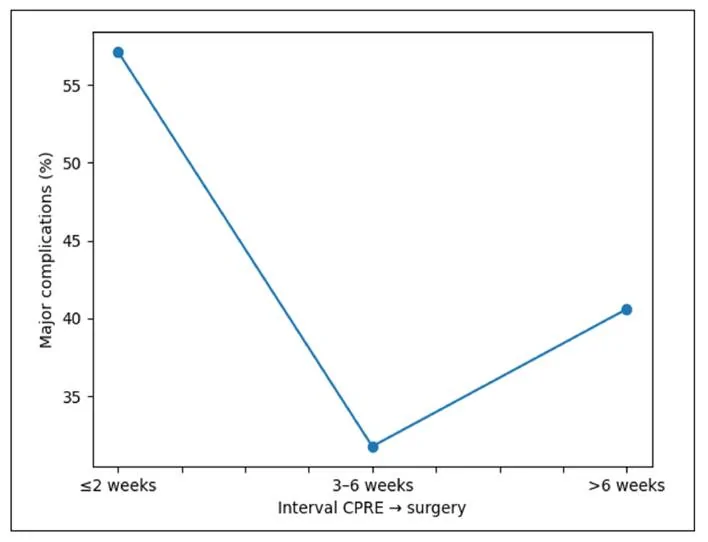
Major complications according to surgical timing in individuals undergoing pancreatoduodenectomy following endoscopic retrograde cholangiopancreatography.

**FIGURE 2:**
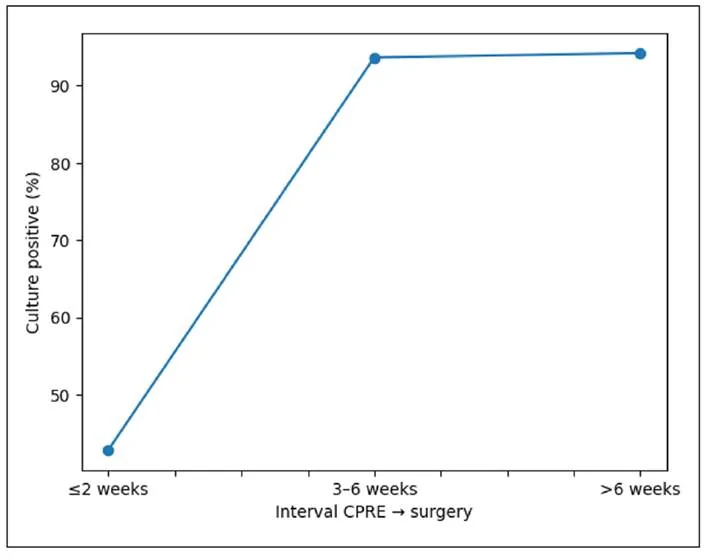
Bile culture positivity according to surgical timing in individuals undergoing pancreatoduodenectomy following endoscopic retrograde cholangiopancreatography.

**FIGURE 3:**
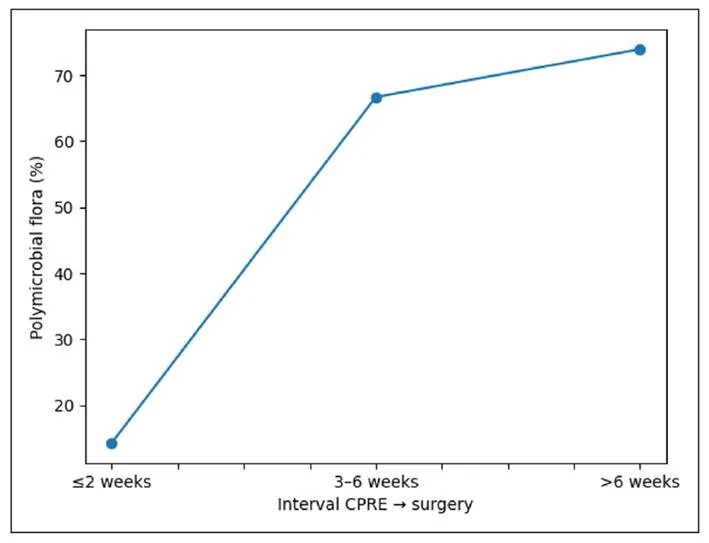
Polymicrobial floral presence according to surgical timing in individuals undergoing pancreatoduodenectomy following endoscopic retrograde cholangiopancreatography.

### Relationship between surgical timing and postoperative mortality

When patients undergoing surgery between 3-6 weeks were compared with all other intervals, 90-day mortality was significantly lower in the intermediate interval group (9.7% vs 23.7%, *p*=0.041). Patients operated outside the 3-6 week interval had a 2.9-fold increased risk of death within 90 days (OR 2.9, 95% CI 1.07-7.83) (Table 5).

**TABLE 5:** Multivariable logistic regression analysis for predictors of 90-day mortality in individuals undergoing pancreatoduodenectomy following preoperative biliary drainage.

Variable	Adjusted OR	95%CI	***P*-value**
Surgery timing (3-6 weeks)	0.08	0.01 - 0.85	0.036
Age (years)	0.99	0.91 - 1.08	0.764
Male sex	2.80	0.56 - 13.98	0.210
BMI (kg/m²)	0.76	0.60 - 0.96	0.024
ASA score	0.27	0.04 - 1.77	0.173
ECOG performance status	3.14	0.89 - 11.04	0.075
Tumor stage	1.78	0.86 - 3.70	0.122
Tumor histology	1.31	0.62 - 2.80	0.480

Outcome: 90-day mortality. Model adjusted for age, sex, body mass index, ASA score, ECOG performance status, tumor stage, and histology. OR: odds ratio. CI: confidence interval. BMI: body mass index. ASA: American Society of Anesthesiologists physical status classification. ECOG: Eastern Cooperative Oncology Group performance status.

## DISCUSSION

### Principal findings

In this retrospective cohort study evaluating the impact of surgical timing after preoperative biliary drainage, we observed that the interval between biliary decompression and pancreatoduodenectomy is independently associated with postoperative outcomes. Specifically, surgery performed between 3 and 6 weeks after drainage was associated with the lowest rates of major complications and 90-day mortality, whereas both earlier and more delayed interventions were associated with worse outcomes. Importantly, this association persisted after adjustment for relevant clinical and oncological variables, suggesting that surgical timing may represent an independent and modifiable factor influencing perioperative risk.

### Interpretation of findings and biological plausibility

Our findings support the existence of a time-dependent balance between two competing pathophysiological processes triggered by biliary drainage. On one hand, early surgery following ERCP may be adversely affected by procedure-related inflammation. Endoscopic manipulation induces local tissue edema, inflammatory infiltration, and microvascular changes within the hepatoduodenal ligament and periportal structures^10^ ^,^ ^11^. These alterations may increase operative difficulty, impair tissue handling, and predispose to postoperative complications. Although the precise duration of this inflammatory phase is not fully defined, clinical data suggest that its impact may extend beyond the immediate post-procedural period^6^ ^,^ ^12^.

On the other hand, delayed surgery exposes patients to progressive bacterial colonization of the biliary tree. Biliary stents rapidly develop biofilms that facilitate polymicrobial growth and increase bacterial load over time^13^ ^,^ ^14^. In the present study, the prevalence of bacteriobilia exceeded 90% beyond three weeks, with a marked increase in polymicrobial contamination. While colonization does not invariably translate into clinical infection, it may contribute to intraoperative contamination and increase susceptibility to surgical site and intra-abdominal infections^15^ ^,^ ^16^.

The observed superiority of the 3-6 week interval likely reflects a window in which post-procedural inflammation has subsided sufficiently to facilitate safer surgical dissection, while the detrimental effects of prolonged bacterial colonization and biofilm maturation have not yet fully translated into clinically significant morbidity. This conceptual framework provides a biologically plausible explanation for our findings and may help reconcile previously conflicting results in the literature.

### Comparison with existing literature

The optimal timing of surgery following preoperative biliary drainage remains controversial, and prior studies have yielded inconsistent conclusions. Some investigations have suggested that early surgery may reduce infectious complications by limiting stent dwell time and bacterial colonization, whereas others have reported increased morbidity associated with surgery performed shortly after ERCP, potentially due to procedure-related inflammation^6^ ^,^ ^12^.

Our findings are consistent with studies demonstrating increased complications with very early surgery, including those reporting higher rates of infectious morbidity when pancreatoduodenectomy is performed within two weeks of ERCP^6^. Conversely, while prolonged delays have been advocated to allow recovery from jaundice and improvement in physiological status, our results suggest that excessive delay may be associated with increased risk, likely mediated by progressive biliary colonization.

Recent studies have begun to emphasize the importance of timing as a key determinant of outcomes. Shin et al.^17^ reported that excessively short intervals between drainage and surgery may be associated with increased major morbidity, whereas intermediate timing may provide a more favorable balance between surgical safety and oncological outcomes. Similarly, Sahora et al.^18^ suggested that preoperative biliary drainage itself may not increase major complications, highlighting instead the importance of patient selection and perioperative management. Satoh et al.^19^ further emphasized that the management and duration of biliary drainage are critical factors influencing perioperative outcomes. In contrast, the meta-analysis by Moole et al.^20^ underscored the ongoing controversy, demonstrating that although biliary drainage may improve physiological derangements, it does not consistently translate into improved surgical outcomes.

Importantly, our study contributes to the literature by integrating clinical outcomes with microbiological data, thereby providing a mechanistic link between timing, bacteriobilia, and postoperative morbidity. This dual perspective supports a more nuanced interpretation of timing, moving beyond a binary “early versus delayed” paradigm toward the identification of an optimal therapeutic window.

### Clinical implications

These findings have direct implications for surgical planning in patients requiring preoperative biliary drainage. In clinical practice, the timing of pancreatoduodenectomy is often influenced by logistical factors, referral patterns, and patient optimization^17^ ^,^ ^21^ ^,^ ^22^. However, our results suggest that surgical timing should be considered an active component of perioperative strategy rather than a passive consequence of scheduling.

When preoperative biliary drainage is indicated, scheduling surgery within a 3-6 week interval may represent a reasonable target in the absence of contraindications. Early surgery within the first two weeks after ERCP should be approached with caution, particularly in the absence of urgent indications, given the potential impact of procedure-related inflammation^6^ ^,^ ^12^. Conversely, prolonged delays beyond six weeks should be avoided when feasible, as they may increase exposure to stent-related complications and bacterial colonization^13^ ^,^ ^14^.

Interestingly, although the 3-6 week group presented high rates of bacteriobilia, this did not translate into worse postoperative outcomes. This finding suggests that the mere presence of bacteriobilia may be less clinically relevant than specific microbiological characteristics, such as polymicrobial contamination or the presence of more virulent or resistant organisms. In this context, our group previously demonstrated that polymicrobial bacteriobilia was independently associated with increased 60- and 90-day mortality after pancreatoduodenectomy, whereas preoperative ERCP itself showed a protective effect in multivariate analysis^23^. These findings reinforce the hypothesis that the microbiological profile and adequacy of perioperative management may be more important determinants of outcomes than bacteriobilia alone.

Nevertheless, timing decisions should remain individualized. Factors such as severity of jaundice, presence of cholangitis, nutritional status, need for neoadjuvant therapy, and institutional logistics must be taken into consideration. Rather than representing a rigid threshold, the 3-6 week interval should be interpreted as a clinically meaningful reference range within a broader patient-centered decision-making framework.

### Additional findings

An additional observation in our study was the association between higher body mass index and lower postoperative mortality, consistent with the so-called “obesity paradox” described in other surgical populations^24^. This finding may reflect the adverse impact of cancer-related cachexia and reduced physiological reserve in underweight patients^25^. However, given the observational nature of the study, this result should be interpreted cautiously and warrants further investigation.

The postoperative mortality observed in this cohort (14.6%) was higher than that reported by many contemporary high-volume centers, but remains consistent with outcomes described in heterogeneous or lower-volume healthcare settings. A nationwide analysis from the Brazilian public health system reported mortality rates of approximately 17% in low-volume institutions, compared with 8% in higher-volume centers, underscoring the impact of institutional experience and resource availability on postoperative outcomes^26^. However, these findings should be interpreted within the context of a tertiary public referral hospital in a middle-income country, which receives a high proportion of complex and delayed referrals. Previous data from our institution demonstrated substantial barriers in access to specialized biliopancreatic care, including delays in referral pathways, limited availability of advanced imaging and ERCP, and socioeconomic vulnerability of the patient population^27^. Moreover, part of the study period overlapped with the COVID-19 pandemic, which significantly disrupted pancreatic surgical activity worldwide and may have negatively affected perioperative care and treatment timing^28^. Finally, endoscopic management strategies evolved throughout the study period, including the progressive adoption of metallic stents in more recent years, which may also have influenced outcomes over time.

### Limitations

Several limitations should be acknowledged. First, this was a retrospective single-center study, which may limit generalizability and introduce selection bias. In particular, the timing of surgery was not randomized and was influenced by clinical and logistical factors, raising the possibility of residual confounding despite multivariable adjustment. Second, the number of patients undergoing surgery within two weeks after drainage was relatively small, which may limit the precision of estimates for this subgroup. Third, microbiological data were not available for all patients, potentially introducing sampling bias in the analysis of bacteriobilia. Another limitation is the incomplete documentation of biliary stent type in a subset of patients, which limited subgroup analyses to cases with identifiable devices. The small sample size of the ≤2-week group may have reduced statistical stability and limited the reliability of subgroup comparisons, particularly for low-frequency outcomes, while also increasing the risk of type I error. The long inclusion period should also be considered when interpreting these findings, as perioperative management, surgical expertise, and endoscopic practices evolved over time. In particular, endoscopic management strategies changed throughout the study period, including the progressive adoption of metallic biliary stents in more recent years, while advances in perioperative care and increasing institutional experience may also have influenced postoperative outcomes over time. Finally, the wide variability in time intervals between drainage and surgery reflects real-world practice but may also introduce heterogeneity that is difficult to fully account for in observational analyses.

## CONCLUSION

The interval between preoperative biliary drainage and pancreatoduodenectomy appears to be an important determinant of postoperative outcomes. In this cohort, an intermediate interval of 3-6 weeks was associated with the lowest morbidity and mortality, suggesting the existence of an optimal window that balances procedure-related inflammation and progressive biliary colonization. These findings support the consideration of surgical timing as a modifiable factor in perioperative management. Prospective studies are warranted to validate these results and to further refine timing strategies in patients undergoing pancreatoduodenectomy.

## Data Availability

data-available-upon-request
